# Women's experience of colposcopy: a qualitative investigation

**DOI:** 10.1186/1472-6874-11-11

**Published:** 2011-04-13

**Authors:** Dawn R Swancutt, Sheila M Greenfield, David M Luesley, Sue Wilson

**Affiliations:** 1 Primary Care Clinical Sciences, The University of Birmingham, Birmingham, UK; 2 Pan Birmingham Gynaecological Cancer Centre, City Hospital, Birmingham, UK

## Abstract

**Background:**

The last comprehensive investigation of women's experience of the colposcopy service in the UK was conducted in the 1980's. It highlighted women's anxiety and lack of information, resulting in recommendations for improvements. Since then the colposcopy service has changed substantially. It is therefore time to re-visit women's experience of this service and reflect upon the success of service changes in improving experience and reducing anxiety. The aim of this study was to investigate women's experience of being referred for, and attending, colposcopy appointments, and identify potential service improvements.

**Methods:**

Qualitative in-depth interviews were conducted with 18 women newly referred for colposcopy in the West Midlands, UK. The interviews were designed to elicit the experience of colposcopy from the patients' perspective.

**Results:**

The eight emerging themes were catogised as three overarching concepts, which were: feelings of emotional reaction, choices being accommodated and time delays. Women felt very apprehensive before their appointment, but when attending, appreciated being consulted about their preferences. Delays in referral and feeling 'rushed' by staff impacted negatively on women's experience.

**Conclusions:**

Service changes in information provision and increased respect for dignity seem to have improved the experience that women have of colposcopy, however, this does not appear to have translated into decreased anxiety. Women still have strong emotional reactions to being referred for, and attending, colposcopy appointments. Staff taking time to explain the diagnosis fully, and discuss their preferences about aspects of their consultation can alleviate their anxiety.

## Background

For over 20 years the UK NHS has run a call-recall programme, systematically inviting women to attend for cervical screening. Annual figures indicate that of those screened over 100,000 women are referred for further investigation consisting of colposcopic examination, yet only a minority of those attending colposcopy go on to receive treatment [1]. Consequently, every year many thousands of women are attending appointments at which they may not require any further medical treatment, and will be discharged. Although this is fundamentally unavoidable in any screening system, for cervical screening it brings an added psychological burden for two reasons; 1. The location of the cervix inevitably means that the investigation is necessarily intimate, and 2. Women attending screening are mainly asymptomatic, thus the referral to colposcopy introduces the idea of medical disease to them, which they have no measure of (no lump, pelvic bleeding or visual difference they can see). It is therefore not surprising that referral for colposcopy is associated with high anxiety levels and psychological distress amongst women.

In the late 1980's a comprehensive investigation was conducted into patient's views of referral through the screening programme [2]. This study made a number of recommendations for service improvement that were based upon the observations made by service users. To alleviate anxiety and provide more information, leaflets were provided at the time of referral. Additional changes such as locking the door to respect patient dignity and providing visual stimulus to give an alternative focus were also proposed. Many of the suggestions, but not all, have since been adopted into routine practice.

In more recent years the colposcopy service has undergone many clinical changes, including the introduction of nurse colposcopists, changes in procedures and developments in treatment methods. Whereas some aspects of women's experience of colposcopy, such as information provision [3-5], anxiety [6,6-8] and the relationship between them [9], have been studied, women's experience of the current colposcopy service as a whole has not been investigated. This paper therefore explores the experience that women have of current colposcopy services and reflects upon whether any further service improvements may be required.

## Methods

In-depth qualitative interviews were conducted with women newly referred to the colposcopy service. The interviews were mainly carried out at colposcopy clinics in two NHS Trusts in the West Midlands; one was a tertiary referral centre, whereas the other was located within a recently constructed outpatient facility serving a particularly diverse ethnic population. Ethical approval was confirmed by South Birmingham Local Research Ethics Committee (Ref: 06/Q2707/13) before the study commenced. Participants were purposively selected to achieve variation in age, referral reason, ethnicity and socio-economic situation. Women were posted details of the study before they attended for their colposcopy appointment and were invited to return a reply slip if they were interested in participation.

Interviews were conducted when women came out of their colposcopy appointment in a private room in the colposcopy clinic, or were arranged to be conducted later at the patient's home. The interviews were semi-structured, following a topic guide, to elicit women's experience of referral to, and attendance at, their colposcopy appointment. The topic guide was informed by current literature regarding barriers to attendance and earlier qualitative reporting of women's views; it aimed to raise questions of a general nature thus providing the opportunity for women to explain their experiences and emphasise what they found important. The participants were reminded of the purpose of the interview and their consent was documented before the interview began.

Interviews were recorded, with the participants' permission, and transcribed verbatim. All interviews were conducted and transcribed by the same researcher (DS). The Framework method of analysis was utilised [10]. This method involved coding and tabulating data, followed by between-case and within-case comparison to identify the variety and frequency of experience for each theme, investigate whether typologies existed and look for explanatory accounts for strongly positive or negative experiences. Three of the authors each independently read three transcripts to test the validity of the emerging thematic framework, which was then revised and applied to all interview data. This is part of a wider study, which employed a mixed-methods approach to better understand women's experience and preferences for colposcopy [11].

## Results

Of the 63 women invited to participate in the study, 21 (33%) agreed to be interviewed. Eighteen of these were interviewed, at which point saturation was achieved (no further information was being generated), therefore study invitations ceased. Interviewees' characteristics are detailed in Table 1.

**Table 1:** Characteristics of interviewees

	Demographic information			Family situation		Colposcopy appointment details		
**Interview No**.	**Age**	**Ethnicity**	**Occupation**	**Partnership status**	**No. children**	**Ist Colposcopy**	**Received treatment**	**Interview location**

1	32	Jamaican	Carer	Married	2	Yes	No	Site 1
2	43	Asian	Student nurse	Single	0	No	No	Site 1
3	59	White	Accountancy asst.	Married	2	No	In past	Site 1
4	30	Black British	Council child care officer	Married	1	Yes	No	Site 1
5	39	White	Carer	Couple	0	No	No	Site 1
6	19	White	Student - biosciences	Boyfriend	0	Yes	No	Site 1
7	56	White	Retired	Married	0	No	In past	Site 1
8	30	Irish	Housewife	Single	3	Yes	No	Site 1
9	23	Gambian	Student	Married	1	Yes	No	Site 1
10	36	Sudanese	Doctor	Married	3	Yes	Yes	Home (Site 1 patient)
11	32	Black African	Civil Engineer	Single	0	Yes	No	Site 1
12	27	White	Student - NVQ childcare	Single	1	No	No	Site 2
13	39	White	Support worker & carer	Single	2	Yes	No	Site 2
14	23	White	Student - English	Single	0	Yes	No	Site 2
15	40	White	Housewife	Married	5	Yes	No	Site 2
16	33	Filipino	Nurse	Married	3+pregnant	Yes	No	Site 2
17	30	White	Community worker	Married	1	No	Yes	Home (Site 2 patient)
18	23	White	Office worker	Single	1	Yes	No	Home (Site 2 patient)

**Figure 1:**
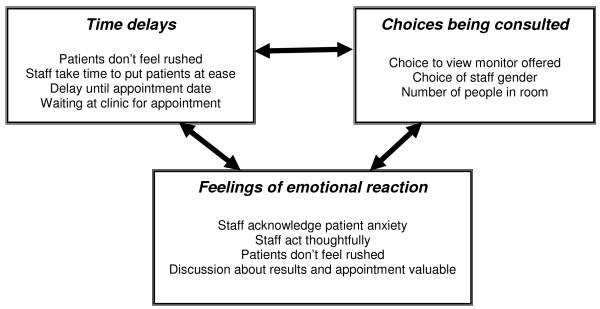
**The relationship between overarching themes of women's experience of colposcopy**.

Eight main themes emerged from the interviews: motivation to attend screening, emotional reactions, family and responsibility, procedural issues, information processing, information sources, outlook, and improvement suggestions. These themes fell within three broad headings; feelings of emotional reaction, choices being consulted and time delays. Though not mutually exclusive, these divisions encapsulate the main issues that women raised; the close relationship between them is illustrated with some examples in Figure 1. The themes that emerged from interviews with women attending colposcopy are presented under these three broad headings.

### Feelings

Many women made comments indicating that they found the experience of being referred for, and attending, a colposcopy appointment emotional. The emotions women recounted were of two types; negative reactions (fear, anxiety, worry, apprehension, embarrassment) and positive reactions (relief, satisfaction, reassurance, that they were glad and felt relaxed).

The most commonly conveyed emotion was anxiety, with distress and embarrassment also expressed strongly. Negative emotions were described in two ways; firstly in relation to what would happen during the appointment, indicating a concern about physical pain or the embarrassment of being exposed to strangers. Secondly, in connection with the outcome of the investigation and what the results meant. The fear of cancer was not often explicitly mentioned but was implicit in many statements of concern for the future.

*"I mentioned it with my partner, again, trying to keep my anxiety, try and keep a lid on my anxiety, erm, not wannin to sort of really overly worry him. The days leading up to coming to the appointment I was a little bit more pre-occupied with, you know, my thoughts and feelings about, you know, what they may find." *Interviewee #4

Women acknowledged the value of positive staff attitude in managing their anxiety and emotional reactions to the situation, but made two improvement suggestions. Firstly, that staff should remember that although it is an everyday job to them, for patients, even those who have attended before, it is an unusual event and because it is so personal they may be very anxious and embarrassed.

*"I know for the nurses who do it an the staff that do it it's a regular occurrence an they probably do 20 a day, but when you're the patient it's a one off for you, an it's embarrassin ...for them it's a job, but when you're the other end, it's quite nerve rackin, it's, it's quite upsettin, it's embarrassin, but no I think, no the staff ave bin good, they're very patient, they're very calm, very reassuring." *Interviewee #7

Whilst explaining her embarrassment this woman praised the reaction of the staff towards her and their behaviour. Secondly, if they become distressed they would like their distress acknowledged and to be treated personally, or cared for. This may be summed up as offering a thoughtful staff attitude.

*"Only that they need in some way ...let women know that it may well be distressing, cause it's not alluded to anywhere in the leaflets, the nurses don't say it...it wouldn't, cost much or y'know, you wouldn't have to do much to make the experience a little bit more sensitive" *Interviewee #17

Many women described how the attitude of the staff was highly important in influencing their experience. The friendliness and reassurance that staff offered was valued as was their clear communication throughout the appointment. A positive staff attitude seemed to alleviate emotional anxiety.

All of the women interviewed appreciated the discussion they had with the colposcopist at the beginning of their appointment for resolving their concerns and misunderstandings.

*"They went through all the details that I need to know, which was very reassuring... so that like, that calmed me down a lot because I was able to talk before the whole procedure, I was just so glad it wasn't straight in there." *Interviewee #8

The level of information required varied widely between individuals and emphasised the need for staff to personalise the delivery of information to each woman according to her own personal needs. In addition, literacy and comprehension levels varied, therefore the wording used by staff needed to be carefully tailored.

*"But some of the words she was talking I didn't, have the meaning [did not understand]" *Interviewee #1

One woman explained how although she had attended appointments previously, she only really understood the explanation from the colposcopist she had just seen.

*"I mean obviously today, and the last time I can remember coming y'know they do explain things to you but I know, I think today's probably the, y'know the best, sort of explanation I've had of what it all is, an what it means" *Interviewee #12

The time spent discussing what the results meant, and what would happen next, was also much appreciated, as it helped women to better understand what was going on and gave them a vocabulary and context in which to describe their condition. Thus discussion alleviated anxiety for many women.

### Choice

Choice was a second important concept. Within the consultation there are a number of areas where women's preferences may be sought. This issue was highlighted by the difference in practice between the two clinics in their approach to the monitor used to enable magnified visualisation of the cervix. Some women were disturbed and offended when the use of the monitor was not explained and they were not asked about their viewing preference.

*"Improve: take the telly away, or turn it, give them an option, d'you wannna watch it or not, y'know what I mean. Put it away from ya, if your partner wants to watch it let him watch it." *Interviewee #15

In contrast, at the clinic where viewing the procedure was discussed in advance, women were generally pleased to be able to have the choice of viewing the procedure.

*"It's good if you want to, wanna see what's going on...but you've got the option to look away, and the nurse said that she would erm, lower my head if I didn't wanna see anything." *Interviewee #6

This implies it is the approach taken, and offer of a choice, that is important for women.

Many women commented upon the thoughtfulness of staff and small details that made them feel as though they were treated personally. However, the converse was also true and women expressed discomfort where introductions or choices were not clearly verbalised by staff. A number of points were raised such as; introduction, explanations of who was in the room and why, explanation of the monitor, and being asked their choice in viewing. Ensuring these points are covered added to the sense of being 'cared for' and not rushed through the appointment.

*"I think it was really good all round, I felt relaxed and I was told what was going on so I wasn't thinking oh what's happening here or what happens if I do this... because I was told about what could happen afterwards as well, after I leave so...I felt I was looked after." *Interviewee #6

Another matter raised was that of the gender of the colposcopist. Whilst a number of women expressed a preference for the gender of the colposcopist they saw, only one caused their appointment to proceed differently because of it. When this woman found that a male colposcopist was to conduct the procedure, she chose the alternative that was offered of having a female instead. A point raised was that although it may be impractical to offer women a choice of colposcopists' gender, it would be helpful to know in advance of the appointment, rather than be surprised on arrival.

*"When they send the appointment out to you they should inform you of the sex of the practitioner that's going to be carrying out, which is quite an intimate test on you. Erm, that you can have that, that sort of erm, self-preparation" *Interviewee #4

The final issue regarding choice was that of the number of people in the examination room. Women reported that the presence of many people, up to four, in the room caused embarrassment during such an intimate examination.

*"There was two nurses, an Asian woman and the docta, all there lookin' an I'm thinkin' oh my God, they're all lookin at me privates." *Interviewee #15

Initially, women may have been told who was in the room and why, but may have forgotten. On a number of occasions they said afterwards that they did not know it was necessary to have so many in the room.

The issue of choice includes the way that information is conveyed. Women described being part of decision-making, and being consulted on preferences and choices, as important in their experience of this examination and procedure.

### Time

Time has already been mentioned with regard to taking time for discussion, explanation, offering choices and care and reassurance during the appointment. Not feeling rushed during their appointment was described as positive for women. Taking time to counsel women and describe what to expect and how it might feel were much appreciated.

*"They were ever so chatty and brilliant, sort of throughout, but not at all in an unnatural or patronising way, that seems sort of, they must be so good at it from having some much experience...then I wasn't forced to get up or... rushed out or that I had to just get straight up, it was really good...and their understanding of, sort of, that it might be a bit uncomfortable afterwards, yeh, they were very good." *Interviewee #14

Time related factors affected experience through waiting times and the feeling of being rushed because others were waiting. Some newly referred women described how concerned they felt when they found that they had a long wait until their colposcopy appointment, or where their GP delayed the referral process.

*"Just the delay, I think you know, it is a very sensitive part of, of medicine you know, women's can you know, need to, because for my experience I was bleeding a lot. This really you know, affect my relationship with, with my husband. Ya, it's really, I'm bleeding you know make me very stressful. So if, if we had you know, these col'oscopy a few months earlier, it would be much better." *Interviewee #10

The time between being informed of an abnormality and having the colposcopy appointment was described as a highly anxious time. Not being clear about the seriousness of their condition exacerbated women's anxiety during this period. Many of the most apprehensive women contacted their GP or practice nurse for advice and information; but became frustrated if they found there had been delays by the GP in referring them to the colposcopy services.

*"Well it felt like months, but I think it was nine weeks altogether, so of course I was actually phoning the hospital here, but because my doctor hadn't referred me...they wouldn't give me an appointment." *Interviewee #8

Most women spoke to friends and family for support. One woman even chose to go for a private appointment, rather than wait, because of her level of concern.

*"Because I was quite anxious about the results ... the results that came back said that I had the highest level of ... pre-cancerous cell changes so, I just wanted to get on with things, just, rang up and found out that if I went private I could get an appointment in like 5 days, rather than waiting 5-6 weeks or whatever it was." *Interviewee #17

Finally, once at the clinic for their appointment, there was a preference to be seen promptly because women experienced high anxiety immediately before the appointment. For some women, a busy waiting area engendered the feeling of being hurried or rushed through their appointment resulting in them feeling reluctant to ask questions.

*"They were obviously understaffed. Maybe they could just look into either only puttin' two slots an hour. Yeh okay would mean people wait longer but at least they wouldn't have to wait as long in the clinic. The receptionist was obviously struggling to get them all out, through, but I would've thought once she'd done her bit there would have been a hell of a long wait." *Interviewee #18

Although it is impossible to tailor each appointment to each woman's needs without limit, those who did not feel rushed commented upon the benefit to their experience. Women valued the feeling that time and care is taken during their appointment.

## Discussion

The principal findings from the interview data were that women's experience was mainly influenced by the three overarching concepts; feelings, choices and time. These concepts were closely related, for example, women not feeling rushed during their appointment and staff taking time to explain the procedure to them, or staff asking women's preferences, made them feel at ease and reduced their anxiety. The largest influence on women's experience seemed to be the empathetic, sensitive and friendly attitude shown by the colposcopy staff; women commonly referred to this.

Women were able to identify practical, and what seemed to be relatively cost-effective, improvement suggestions e.g. being notified the gender of the colposcopist with their appointment letter, improved appointment-scheduling to reduce waiting times in the clinics, the ability to avoid seeing the procedure on the monitor, reducing the number of people in the colposcopy suite during treatment, and the tailoring of information provision to individual women's needs. These suggestions for improvement may not improve clinical outcomes, but may substantially reduce the emotional impact colposcopy has for many women, and improve their experience of this procedure.

This study has updated the work conducted by Posner and Vessey in the late 1980's, and describes the experience that women have of current colposcopy services. It has highlighted that although colposcopy is viewed as a relatively routine procedure by clinicians, women relating their experience describe it as a highly emotional encounter. The considerable distress woman experienced, upon receipt of an abnormal smear result, was acknowledged by Posner and Vessey, and was also found in this study. The theme of emotional reaction was returned to again and again by women as they recounted their reactions to being referred for a colposcopy. In the current study, similar to Posner and Vessey's findings, distress was associated with women not understanding the meaning of the smear test result, and fear that cancer had been detected. Despite interventions to promote information provision, women using the colposcopy service conceptualised health and illness as black and white, with the 'grey' area of CIN progression still being poorly understood.

In keeping with Posner and Vessey's study, this study found that women described a sense of relief, reassurance and gratitude after their appointment was over. These emotions were attributed to the clarification of diagnosis during the appointment, and gaining understanding of how the condition is managed. This study also found that women described positive emotions as a result of attending their appointment, particularly relating to the friendliness of the clinic staff. Comments were made on the thoughtfulness of staff in relation to small issues, such as explaining how to sit on the couch, or introducing who was in the room. Attending to these details added to women's sense of being 'cared for' and 'put at ease'. It is not clear whether these positive emotional responses were a result of increasing women's understanding regarding their condition and treatment options, the attitude of the clinic staff, or a combination of both.

The descriptions women gave of their emotional reaction and confusion over the meaning of smear test results were consistent with the concept of liminality [12] and indicate how being neither healthy nor ill, but between the two, affects women's ability to make sense of their current condition. This confusion intensifies the emotional state which also reduces the ability to take in and retain information when an individual feels that their health is under threat [13].

This study found that, in common with the work of Nugent *et al. *in the early 1990's [6,14], there remains widespread lack of understanding of the meaning of a smear test result or the purpose of colposcopy. Anxiety levels were high before women's appointments, though alleviated where suitable explanations were provided during their consultation. This supports Nugent *et al.'s *suggestion that nurturing a therapeutic relationship to support women's informational and emotional needs might alleviate anxiety. Whilst this study found that those informational and emotional needs were mostly met, there were occasions where women left their appointment still not having a clear grasp of the purpose of the colposcopy, and where they felt emotional needs were overlooked.

Women's attitude towards the magnified view of their cervix, displayed on the monitor during the procedure, corroborated Howson's [15] observations of women's reactions to this sight. Her conclusion that the magnified view turns their cervix into a medicalised object, rather than providing reassurance for women was borne out with this group of women. Women seemed to like the idea of being able to view the procedure when the choice was offered, but did not find the viewing as helpful as they expected it to be.

Qualitative research methods can be criticised for their small sample size and inability to provide generalisable results. However, although only 18 women were interviewed for this study, theme saturation was accomplished, meaning that no new information would be generated by continuation with further interviews. The aim of this study was to represent the range of views or experiences to better understand the topic, rather than collect a demographically representative sample that can be generalised.

## Conclusion

It is clear from the interview findings that women's experience of colposcopy is emotional, with positive and negative feelings ensuing. Anxiety exists before the appointment, but subsequently when the procedure and meaning of the cervical smear test results are explained, many women feel relief. Comparison with the study conducted by Posner and Vessey (1988), and other qualitative work, shows these attitudes remain common in women attending for colposcopy. Despite better written information provision, aimed at reducing anxiety, the emotional reaction women undergo after receiving an abnormal cervical smear test result seems to have changed little over the last 20 years.

The impact of a thoughtful and empathetic staff attitude is highlighted by women's descriptions of the reduced anxiety they felt as a result of staff calmness and reassurance. Women express the benefit of being consulted on their preferences for aspects of the appointment, which illustrates the desire, of many women, to be involved in decision-making during their appointment. This study has found that feelings, time and choices influence each other, and if we assume that because of financial constraints we are limited in our ability to provide more time to each woman, we should therefore focus upon addressing individual women's choices and identifying their preferences. This area of research, concerning women's preferences for aspects of their colposcopy appointments, would benefit from further research, as would work to identify strategies to reduce anxiety between referral and attendance for colposcopy.

## Competing interests

DL is the Editor of the current (2010) colposcopy guidelines.

## Authors' contributions

The idea for this study was developed by SW and DS. All authors contributed to the design of the study. DS conducted data collection and analysis, and completed the initial draft of the paper. All authors have contributed to and approved the final paper.

## Pre-publication history

The pre-publication history for this paper can be accessed here:

http://www.biomedcentral.com/1472-6874/11/11/prepub
